# Dr. C. E. J.

**Published:** 1874-02

**Authors:** 


*Dr. C. E. J.*—We think you are mistaken in supposing that the Professors of all the Medical Colleges use the names of their friends in the Drug and Insurance business to swell their list of matriculants. We think, however, there are some *sharp* enough, and it would be well for all to be on the look out.

